# Supplementary material for: Titers of antibodies against ancestral SARS-CoV-2 correlate with levels of neutralizing antibodies to multiple variants
Source: NPJ Vaccines. 2022 Dec 30;7:174. doi: 10.1038/s41541-022-00586-7 (PMC9801350; doi:10.1038/s41541-022-00586-7)
Supplement: Supplementary file 1 — Supplementary Information [file 41541_2022_586_MOESM1_ESM.pdf]

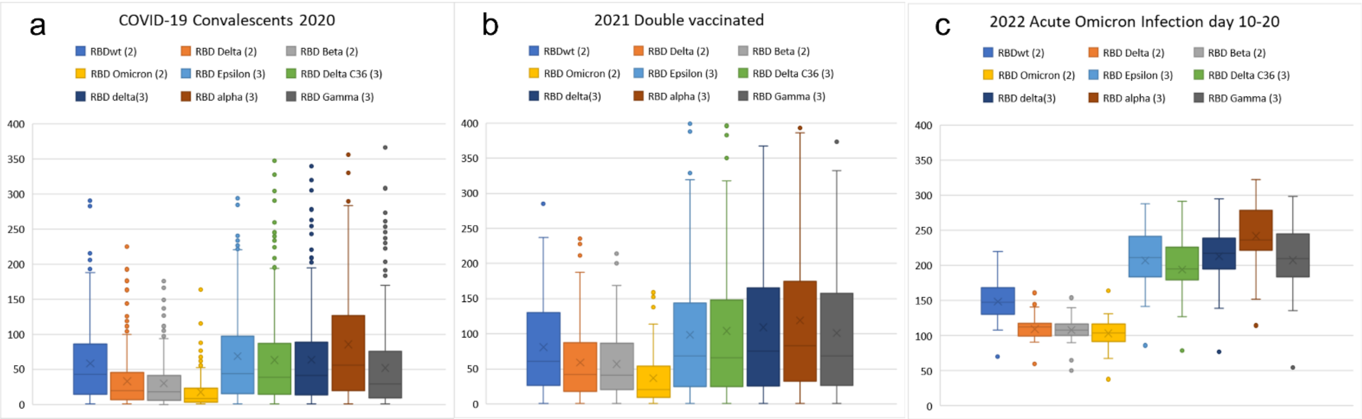

Supplementary Fig. 1: Signal values measured for antibodies to RBDs from SARS-CoV-2 variants.

The bar blots show rMFI of anti-human IgG measured for beads coupled with RBD from indicated SARS-CoV-2 variant. Beads were incubated with serum diluted 1:1000 for 1h and labelled with anti-human IgG. Covid-19 convalescents: sera obtained in 2020 from individuals with mild COVID-19 (n=291). 2021 Double vaccinated: sera obtained in 2021 from individuals who had received two doses of the Pfizer/BioNTech vaccine (n=140). 2022 Acute Omicron BA.1 Infection: sera obtained from healthy double-vaccinated individuals 10-20 days after symptom debut of Omicron BA.1 infection (n=22). Source data are provided as a Source Data file.

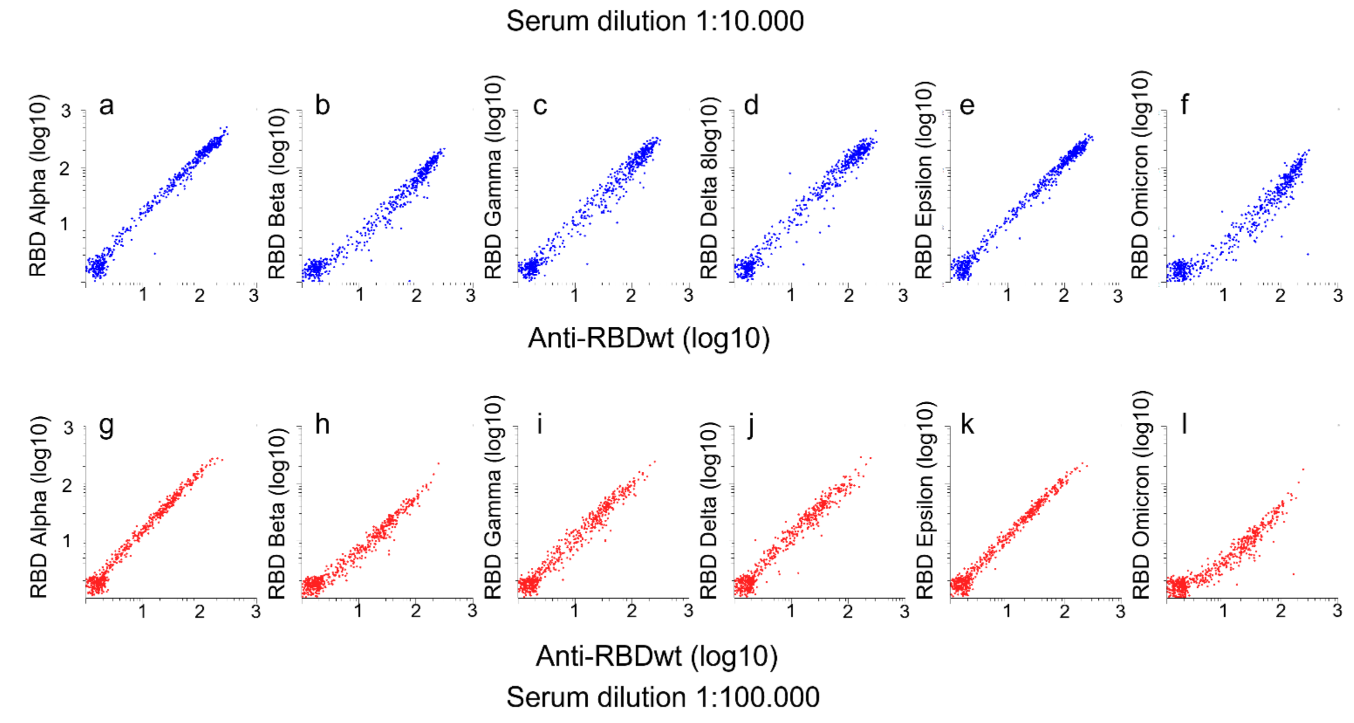

Supplementary Fig.2. Correlation between antibody levels to RBDs from SARS-CoV-2 variants

The dot plots show rMFI for anti-human IgG measured for beads coupled with RBDs from indicated SARS-CoV-2 variant. Each dot corresponds to a different sample. **a-f:** beads were incubated with 550 sera diluted 1:10,000 for 1h prior to labeling with anti-human IgG. **g-l:** beads were incubated with 550 sera diluted 1:100,000 for 1h prior to labeling with anti-human IgG. Source data are provided as a Source Data file.

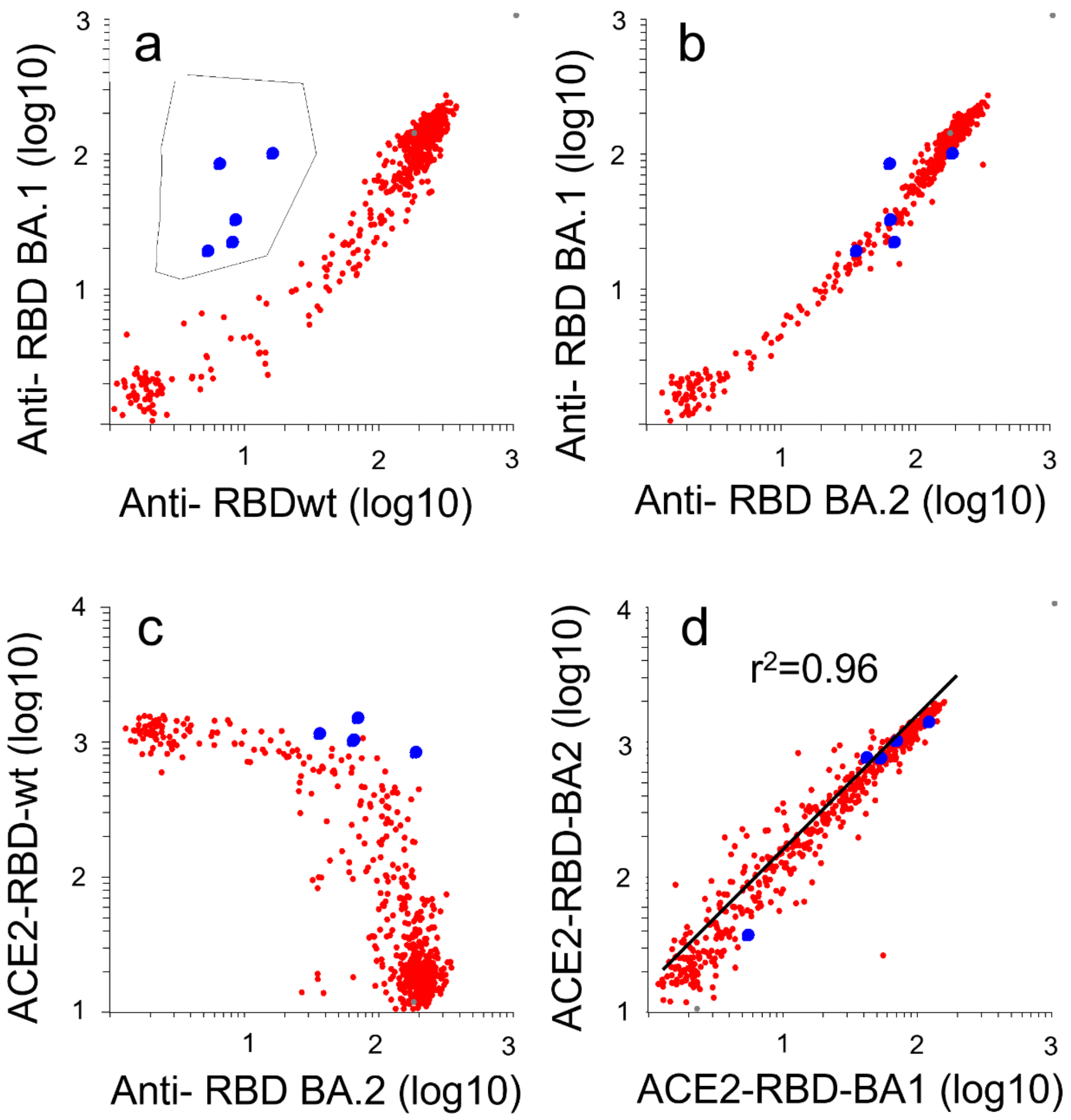

Supplementary Fig.3. Correlation between binding- and neutralizing antibodies to RBD from SARS-CoV-2wt and the Omicron BA.1 and BA.2 variants

The dot plots show binding of IgG or ACE2 to RBD proteins from indicated SARS-CoV-2 variant. Each dot corresponds to a different sample. Bead-based arrays were incubated with serum diluted 1:1000 or 1:100 for measurement of IgG and ACE2-binding, respectively. Blue dots indicate samples with antibodies to RBD from Omicron only. Source data are provided as a Source Data file.

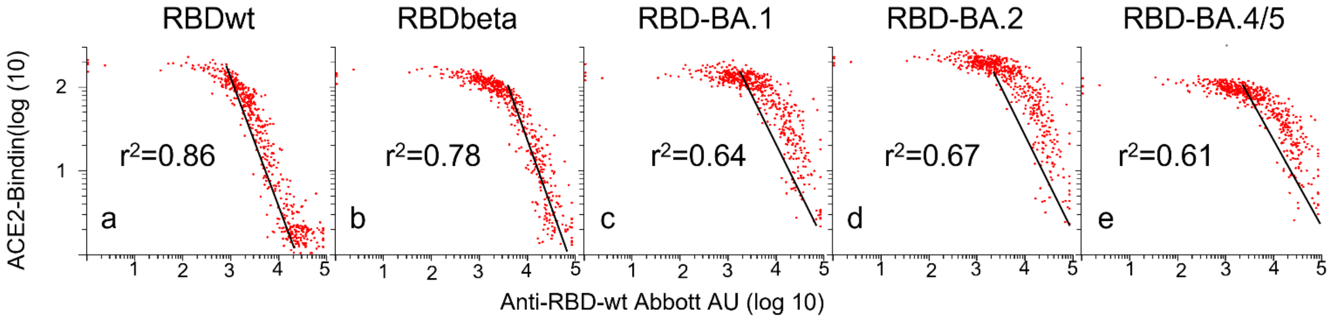

Supplementary Fig.4. Correlation between array-based measurement and the Abbott anti-spike assay.

A total of 528 sera were analyzed in parallel with the Abbott anti-Spike assay and multi-IgG-ACE2-RBD. Each dot in the scatter plots correspond to a different sample. The x-axes show anti-RBDwt in arbitrary units (AU) measured by the Abbott SARS-CoV-2 IgG II Quant assay. The y-axes show binding of ACE2 to RBDs from indicated SARS-CoV-2 variant. Correlation coefficients were determined for anti-RBDwt titers in the range of 1000-10000 AU for ACE2-binding to RBDwt (a) and in the range of 3000-800000 AU for binding to other RBDs (b-e). Source data are provided as a Source Data file.

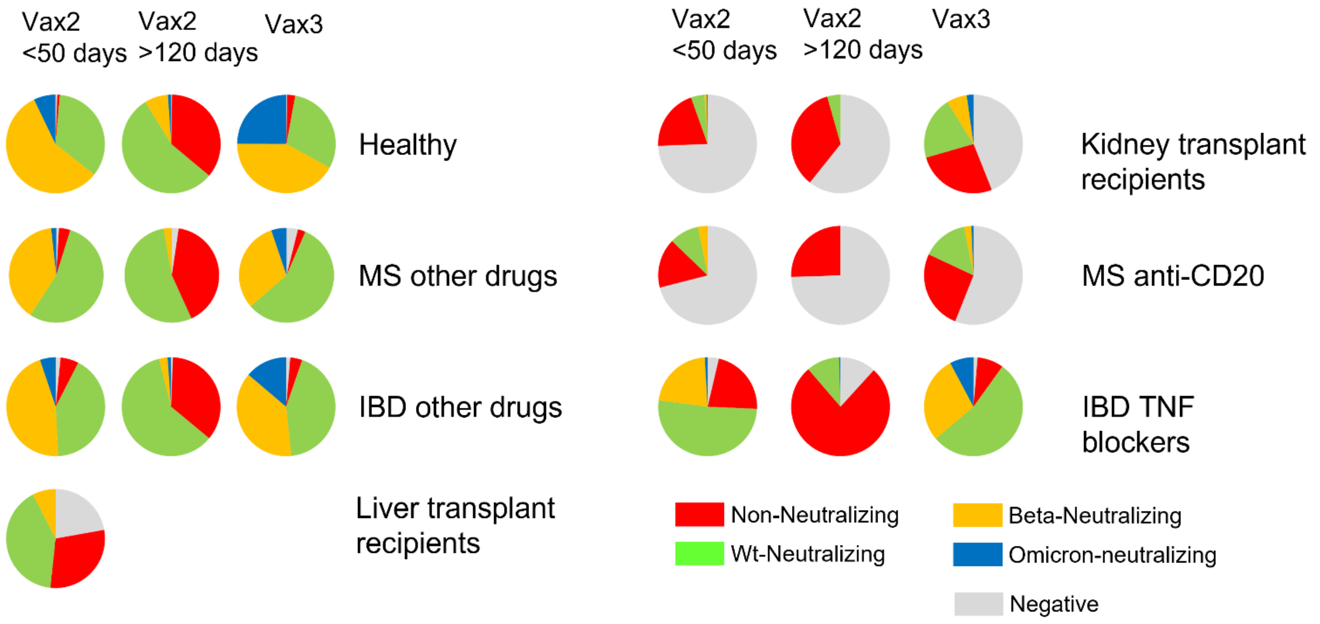

Supplementary Fig. 5. Classification of post-vaccine sera obtained from healthy individuals and patients on immunosuppressive therapy.

The pie charts show frequencies of post-vaccine sera from indicated cohort classified according to group I-IV in Fig. 3a. See legend to Fig. 8. Source data are provided as a Source Data file.

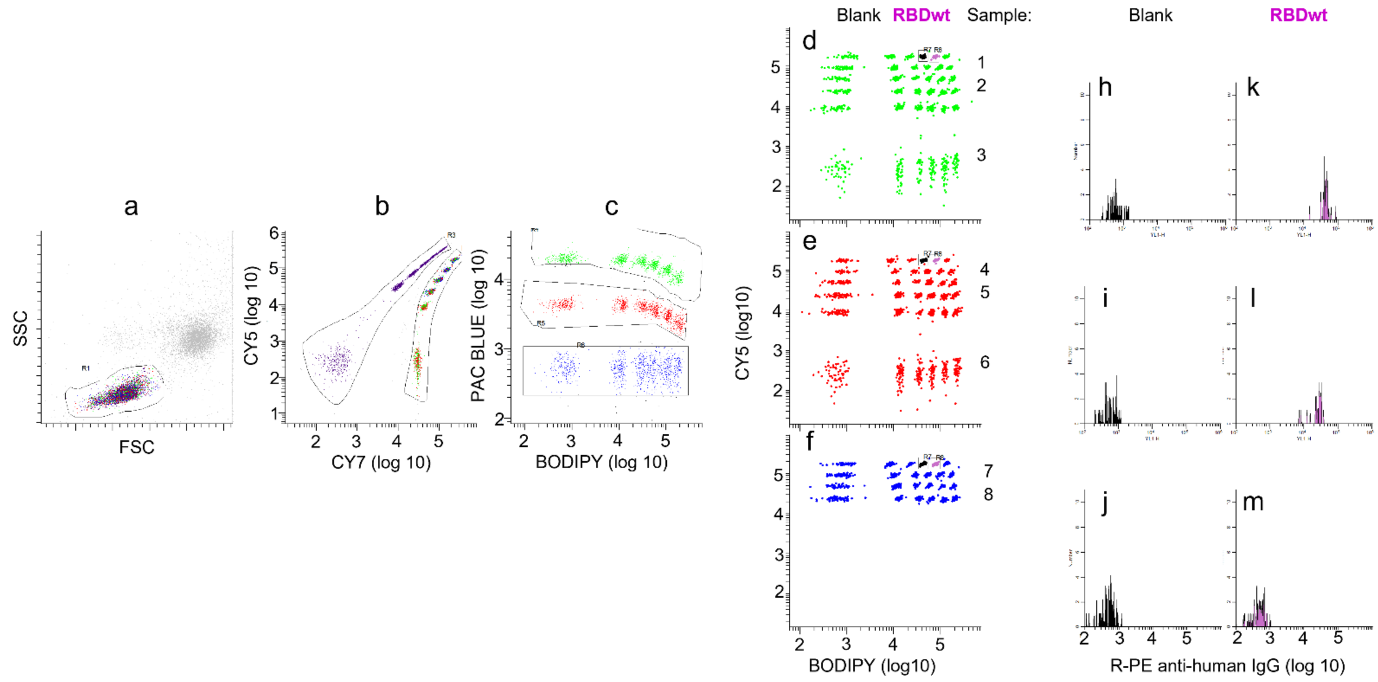

Supplementary Fig. 6. Gating strategy

The dot plots show the gating strategy used for bead-based arrays. a) Forward scatter (FSC) and side scatter were used to discriminate beads with diameters of 6 and 8mm, respectively. The region indicates beads with a diameter of 6µm. B) Cy7 and Cy5 were used to discriminate two arrays with different content of proteins. c: Pacific Blue was used to discriminate subarrays incubated with different samples (see Fig. 1). d-f: Bodipy and Cy5 were used to discriminate individual bead subsets. The multiplexing corresponds to different proteins as well as different samples (see also Fig. 1). h-j: anti-human IgG R-Phycoerythrin fluorescence of microspheres with no virus protein. Each pair of histograms correspond to results obtain with a different sample. k-m: anti-human IgG R-Phycoerythrin fluorescence of microspheres coupled with RBD from SARS-CoV-2wt. Each pair of histograms correspond to results obtained with a different sample. The gating strategy for 8µm beads is the same.

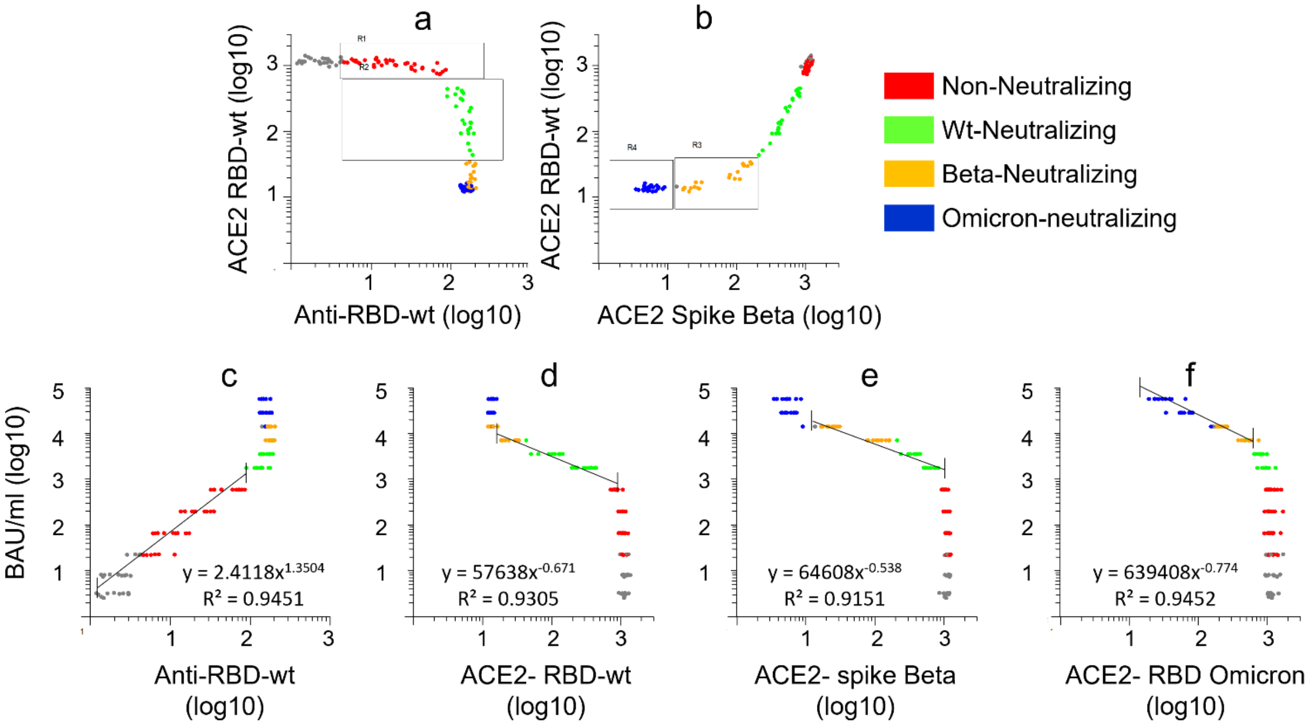

Supplementary Fig. 7 Calculation of BAU/ml

The dot plots show all results obtained with the standard series on 384 well plates analyzed in the period of Feb 14-March 3, 2021. Each dot represents a different sample. **a-b**: see legend to Fig. 5. **c-f:** The y-axes show BAU/ml calculated from serial dilutions of the standard series. The x-axes show binding of IgG to beads coupled with RBDwt (**c**), or ACE2 binding to beads coupled with RBDs or spike proteins as indicated (**d-f**). Values within the ranges of the lines drawn on each plot were used as input in the Excel regression function. The numbers and text within each plot show the formulas returned by Excel and the squared correlation coefficients. Source data are provided as a Source Data file.
